# Botulinum toxin therapy of dystonia

**DOI:** 10.1007/s00702-020-02266-z

**Published:** 2020-10-30

**Authors:** Dirk Dressler, Fereshte Adib Saberi, Raymond L. Rosales

**Affiliations:** 1 Movement Disorders Section, Department of Neurology, Hannover Medical School, Carl-Neuberg-Str. 1, 30625 Hannover, Germany; 2 Department of Neurology and Psychiatry, Neuroscience Institute, University of Santo Tomas Hospital, Manila, Philippines

**Keywords:** Botulinum toxin, Therapy, Dystonia, Treatment strategies

## Abstract

Botulinum toxin (BT) is used to treat a large number of muscle hyperactivity syndromes. Its use in dystonia, however, is still one of the most important indications for BT therapy. When BT is injected into dystonic muscles, it produces a peripheral paresis which is localised, well controllable and follows a distinct and predictable time course of around 3 months. Adverse effects are always transient and usually mild, long-term application is safe. With this profile BT can be used to treat cranial dystonia, cervical dystonia and limb dystonia including writer’s and musician's cramps. The recent introduction of BT high dose therapy also allows to treat more wide-spread dystonia including segmental and generalised dystonia. BT can easily be combined with other anti-dystonic treatments such as deep brain stimulation and intrathecal baclofen application. Best treatment results are obtained when BT therapy is integrated in the multimodal and long-term 'multilayer concept of treatment of dystonia'. The biggest challenge for the future will be to deliver state of the art BT therapy to all dystonia patients in need, regardless of whether they live in developed countries or beyond.

## Introduction

Botulinum toxin (BT) is used to treat a large number of muscle hyperactivity syndromes, disorders of exocrine glands and pain conditions (Truong et al. 2013). Dystonia, however, is still one of the most important indications for BT, both with respect to the amount of BT consumed as well as with respect to the therapeutic impact generated. Hence, this review aims to elucidate the role of BT therapy for the treatment of dystonia.

## Pharmacology of botulinum toxin

### Therapeutic profile

As shown in Table 1, BT can be used in muscles, exocrine glands and pain associated structures. When BT is injected into muscle tissue, its therapeutic effect is a peripheral paresis which is localised, well controllable and follows a distinct and predictable time course. It manifests clinically after few days, reaches its maximum after one to two weeks, is usually stable for 6–12 weeks and then gradually, but completely resolves over several weeks. All adverse effects are also transient. They may be obligate, local and systemic. Systemic adverse effects hardly occur when BT type A drugs are used.

**Table 1:** Therapeutic profile of botulinum toxin drugs

Target tissues
Striate and smooth muscles ('neuromuscular junction')
Exocrine glands
Structures involved in generation, perception or
Transmission of pain
Therapeutic effect
Localised
Extent well controllable
Predictable time course
Fully reversible
Adverse effects
Fully reversible
Obligate adverse effects manageable
Local adverse effects few
Systemic adverse effects:
BT-A: extremely rare
BT-B: frequent anticholinergic adverse effects

### Botulinum toxin drugs

BT drugs are usually based on BT type A, such as onabotulinumtoxinA (ONA, Botox^®^, Allergan, Dublin, Ireland), abobotulinumtoxinA (ABO, Dysport^®^, Ipsen Pharma, Boulogne-Billancourt, France) and incobotulinumtoxinA (INCO, Xeomin^®^, Merz Pharmaceuticals, Frankfurt/M, Germany). RimabotulinumtoxinB (RIMA, Neurobloc^®^/MyoBloc^®^, US WorldMeds, Louisville, KY, USA) is the only BT drug based on BT type B. A large number of additional BT drugs are currently being developed in Asia. Most of them are using ONA as a reference.

The properties of BT type A drugs are similar, but differ considerably from those of the BT type B drug. BT drugs are biologics and as such there are no generics. They can and need to be compared and they may be interchanged in an ongoing therapy. For potency comparisons conversion factors need to be applied. The conversion factor for ONA and INCO is 1:1 (Dressler 2012; Dressler et al. 2012), for ONA/INCO and ABO it is still debated. In summary, ONA is still the reference BT drug, INCO excels with a reduced antigenicity, ABO suffers from a lack of comparability because of its idiosyncratic potency labelling and RIMA has systemic adverse effects and antigenicity problems.

## Basic principles of botulinum toxin therapy

### BT therapy in the multilayer concept of treatment of dystonia

Dystonia is a chronic condition without causal therapy. In most cases, dystonia treatment has to be multimodal and long-term. A recent consensus paper (Dressler et al. 2015) by IAB—Interdisciplinary Working Group for Movement Disorders (Adib Saberi and Dressler 2013) describes this complexity and introduces an algorithm to structuralise it. It is important to bear in mind that BT therapy is only one aspect of the broad spectrum of treatment options for dystonia.

Treatment of dystonia can best be described as a multilayer concept (Table 2). The basal layer includes anti-dystonic therapies. Here, BT therapy is pivotal. It is entirely symptomatic, but its use may change the long term perspective of patients by avoiding the development of complications potentially dominating the clinical picture. Use of BT therapy early in the course of dystonia, therefore, seems advisable. Usually, BT therapy should be complemented by physiotherapy (Barth and Dressler 1993; Dressler and Adib Saberi 2017b). BT therapy can easily be combined with any other anti-dystonic therapy including oral drugs, intrathecal baclofen and peripheral or central surgery including deep brain stimulation.

**Table 2:** The multi-layer concept of comprehensive dystonia therapy

Layer	Description	Modalities
1	Anti-dystonic treatment	Botulinum toxin therapy
		Oral drugs
		Intrathecal baclofen
		Surgical interventions
2	Adjuvant drugs	Analgesics
		Anxiolytics
		Antidepressant
3	Adjuvant treatment	Physiotherapy
4	Adjuvant measures	Social support
		Providing information
		Patient support groups

The second layer includes adjuvant drugs such as analgesics, anxiolytics and Antidepressants. Relaxation exercises can break the vicious circle of motor induction in dystonia.

The third layer consists of adjuvant measures including adequate information for the patient and his family. Patient support groups have a role to play here. Social support gives advice to social benefits available.

### BT treatment algorithms

Treatment algorithms include the set of treatment parameters and the treatment strategies to combine and modify them for adaptation to the individual patient's situation. Recent consensus guidelines give an overview over the algorithms used for BT therapy (Dressler et al. 2020). Treatment parameters include target muscles, target muscle doses, inter-injection intervals, total doses, dose distribution within the target muscle, drug dilutions, guidance techniques, choice of drug etc. Treatment strategies cover treatment initiation, treatment adaptation and reactions to treatment failure. Most recent advances of BT treatment algorithms include the BT high dose therapy (Dressler et al. 2014; Wissel et al. 2017) and the BT short interval therapy (Dressler and Adib Saberi 2017b).

### Dosing

Dosing is the most crucial parameter of BT therapy. It depends on two elements, i.e. the number of target muscles and their BT doses reflecting the degree of their dystonic involvement. Recent consensus guidelines introduce updated dosing tables (Dressler et al. 2020). Indicated dose ranges should be specified by dose modifiers (Table 3) to individualise BT therapy further.

**Table 3:** Dose modifiers for botulinum toxin therapy

Sex	Male	Dose increase
	Female	Dose reduction
Age	Young age	Dose increase
	Old age	Dose reduction
Body weight	High	Dose increase
	Low	Dose reduction
Proportional muscle mass	High	Dose increase
	Low	Dose reduction

### Planning of BT therapy

Planning of BT therapy is based on identification of target muscles and their degree of dystonic involvement. It leads to the development of the individual dosing scheme. Necessary information may be obtained clinically by analysing the dystonic movements or postures and the dystonia-related pain (Siongco et al. 2020). Special electromyography may improve this planning (Dressler 2000). Simultaneous multi-channel electromyography is difficult to control and does not necessarily provide more information than antagonistic recordings. Target muscle selection by estimating muscle hypertrophy is inadequate. The resulting injection scheme is a prediction of the patient's therapeutic response. Subsequent injection series will usually improve the initial injection scheme.

### Intramuscular BT placement

BT placement in the target muscle can be performed clinically by palpation (ideally under voluntary activation of the target muscle), using surface landmarks or by back-tracing the muscle tendons. To identify individual finger muscles repetitive active or passive finger movements may improve palpation. In target muscles, in which this is difficult, such as the iliopsoas or the piriformis muscles, or when selective injection of individual finger muscles is required, the use of guidance techniques may become necessary. Electromyographic guidance uses special injection needles. Simultaneous electric stimulation may be of additional help. Ultrasound guidance is an alternative (Walter and Dressler 2014). It seems to be particularly helpful in children who may be uncooperative and particularly pain sensitive (Berweck et al. 2002).

## Specific indications for botulinum toxin therapy of dystonia

### Cranial dystonias

Cranial dystonias can affect periocular, mandibular and perioral muscles. Rarely, scalp muscles or periauricular muscles are affected (Alonso-Navarro et al. 2007). BT therapy can be used in all of these muscles successfully, either when they occur in isolation or when they occur in various combinations. In periocular dystonia, producing the classical clinical picture of blepharospasm, BT is injected into the orbicularis oculi muscle responsible for eyelid closure (Frueh et al. 1984; Costa et al. 2005). Additional target muscles include the procerus and the corrugator supercilii muscles which form the horizontal and vertical nasal root folds and narrow the eyebrows. They may produce tension, but don't have much influence on eyelid function. The nasalis muscle forms the longitudinal nasal dorsum fold and can be injected when the patient complains of irritation especially when wearing glasses. The frontalis muscle is an accessory eyelid opening muscle and, therefore, should not be injected in blepharospasm contrary to occasional belief. Doses, dilutions and injection points for the treatment of blepharospasm vary considerably, whereas results and adverse effects are surprisingly similar. Adverse effects include ptosis, double vision, lagophthalmus and hematoma. They are rare and transient. Ptosis can almost certainly be avoided by sparing the medial part of the upper eyelid. Some patients with blepharospasm, especially those with progressive supranuclear palsy, have a varying degree of additional apraxia of eyelid opening (Aramideh et al. 1994), i.e. a supranuclear impairment of the eyelid opening mechanism. In those patients additional BT injections close to the rim of the eyelid are helpful (Jankovic 1996). If this doesn't help, a bilateral suspension operation connecting the upper eyelid to the frontalis muscle by a subcutaneous non-resorbable thread is helpful (Dressler et al. 2017). In mild cases a wire spring attached to a spectacle frame can produce similar effects.

In perioral dystonia muscles above the oral orifice should generally be injected with special caution to avoid drooping of the mouth. BT injections into the upper lip may produce paraesthesias for unknown reasons. The risorius muscle can be injected safely 2 cm lateral to the corner of the mouth, whereas injections into the depressor labii inferioris bear the risk of instability of the lower lip.

In mandibular dystonia jaw opening and jaw closing forms can be distinguished (Marsden 1976). Combined activation of opening and closing muscles, however, are not infrequent. Additional jaw movements include jaw protrusion and lateral shifts of the jaw. Jaw closing is caused by activation of the masseter, the temporalis and—to a minor extent—the medial pterygoid muscles. Jaw opening is the result of activation of the lateral pterygoid muscle and the suprahyoid muscles forming the muscular floor of the cavity of the mouth. Protrusion and lateral shifts are caused by the pterygoid muscles, mainly the lateral ones. Whereas treatment of the jaw-closing type produces excellent results with only rare adverse effects, treatment of the jaw opening type is less rewarding. Our experience indicates that BT injections into the pterygoid muscles through the incisura mandibulae together with injections of the suprahyoid muscles seem to work best in this situation. Attempts to inject the lateral and the medial pterygoid muscles selectively cause major technical problems and discomfort for the patient. Local spread and frequent co-activation of both muscles also question the logic of this approach.

### Pharyngolaryngeal dystonia

Tonic or clonic dystonia of the pharynx can produce dysphagia and dyspnoea (Zwirner and Dressler 1995). They can occur spontaneously or in an action-induced fashion. BT injections into the posterior pharynx can easily be placed transorally and are effective. Doses range between 20-40 MU of ONA. Laryngeal dystonia produces the clinical picture of spasmodic dysphonia, either in the adductor form with a strained-strangled voice or in the much less frequent abductor form with hypophonia (Marsden and Sheehy 1982). In adductor forms 2.5–10 MU of ONA are administered into the thyroarytenoid (vocalis) muscle. Unilateral application appears to produce less adverse effects than when the same amount is distributed over both sides. In abductor forms 2.5 to 10 MU of ONA are administered into the posterior cricoarytenoid muscle unilaterally to avoid dyspnoea. BT application can be performed perorally or transcutaneously using electromyographic guidance. The transoral approach allows detection of additional dystonic muscle activities in the pharynx or the larynx and, therefore, seems to be the superior method.

For the patient and for the physician, BT therapy of spasmodic dysphonia represents a highly satisfying indication (Whurr et al. 1998). Practically all patients benefit from BT therapy and the degree of improvement is astonishing. In many cases almost normal speech patterns can be regained. In patients with abductor forms the treatment results are less favourable. Adverse effects include difficulties with swallowing liquids or solid food. BT therapy can also induce weakness of coughing and some pain at the injection side. In treatment of adductor forms hoarseness, breathiness of voice and hypophonia can occur, whilst in treatment of abductor forms dyspnoea may result.

Spasmodic laryngeal dyspnoea describes spontaneously occurring or respiration-induced muscle hyperactivity of laryngeal muscles (Zwirner et al. 1997). This condition is very rare and may influence both, glottic and supraglottic muscles and can also be treated with BT therapy.

### Cervical dystonia

Cervical dystonia induces deviation of the neck and the head. Frequently, shoulder elevation occurs. Whereas the neck can only be flexed and extended on a sagittal plane or frontal plane and rotated on a horizontal plane throughout its entire structure employing numerous interspinal joints, the head can be flexed and extended on a sagittal plane, can be tilted on a frontal plane and can be rotated on a horizontal plane by using the single atlanto-occipital joint with the dens as an additional stabiliser. Head and neck deviations can best be described by the scheme shown in Table 4. The scheme allows an easy and semiquantitative, although comprehensive description of the head and neck deviations elicited by cervical dystonia. Isolated occurrence of head or neck deviations is rare. Most patients suffer from complex combinations of head deviations.

**Table 4:**
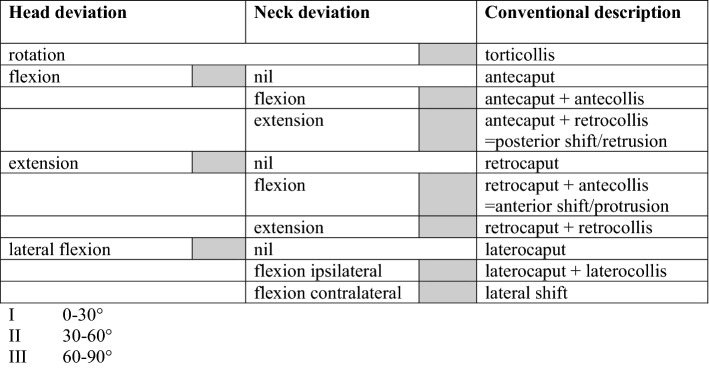
Description of head and neck deviation in cervical dystonia

Head rotation occurs always together with neck rotation. It is caused by activation of the ipsilateral splenius capitis, the contralateral sternocleidomastoid muscle and the ipsilateral trapezius/semispinalis capitis muscle complex. Deep posterior neck muscles arising from the atlas and the axis including the obliquus capitis inferior, the rectus capitis posterior major and the rectus capitis posterior minor muscles are strong ipsilateral head rotators. The levator scapulae muscle is an additional, but weaker ipsilateral head rotator. In head and neck rotation the role of the sternocleidomastoid is often overestimated, whereas the role of the splenius capitis and the deep posterior neck muscles is often underestimated.

Head flexion is caused by activation of the supra- and infrahyoid muscles. Neck flexion is produced by the scalenii and deep anterior neck muscles including the longus colli, the longus capitis and the rectus capitis anterior muscles. Head extension originates from bilateral activation splenius capitis and the deep posterior neck muscles, whereas neck extension is elicited by bilateral activation of the trapezius/semispinalis capitis muscle complex. Lateral head flexion originates from ipsilateral activation of the sternocleidomastoid and the splenius capitis, whereas lateral neck flexion is produced by ipsilateral activation of the scalenii, the levator scapulae and the trapezius/semispinalis capitis muscle complex. Protrusion is the consequence of neck flexion together with head extension and retrusion of neck extension together with head flexion.

Dystonic pain, as the leading complaint in most patients with cervical dystonia, can almost always be markedly reduced by BT therapy. Residual pain may be caused by secondary degenerative processes or by radicular irritation. Head posture can also be improved substantially. Often, patients report the effects of the first BT applications enthusiastically, most likely due to the contrast to the sometimes prolonged period of insufficient treatment. Especially in the treatment of cervical dystonia additional physiotherapy is necessary (Barth and Dressler 1993; Dressler and Adib Saberi 2017b). Certain forms of cervical dystonia respond less favourably to BT therapy. Head flexion and neck flexion are particularly difficult to treat when deep anterior neck muscles are involved, but also alternating types of cervical dystonia may present a therapeutic challenge. In tremor types sometimes reduced BT doses seem to be helpful.

The most common adverse effect of BT therapy of cervical dystonia is dysphagia. Depending on the definition and severity of dysphagia, the effort to search for it and depending whether patients with optimised BT therapy are considered, its frequency varies greatly. Applying current treatment standards certainly less than 5% of patients experience dysphagia constantly after each injection series. Mild dysphagia may be more frequent. Ultrasound guidance does not seem to reduce the dysphagia frequency (Kutschenko et al. 2020). Another adverse effect is head instability, especially due to impaired head and neck extension. When these adverse effects occur their duration is usually limited to one or two weeks. Injection of the scalenii muscles can produce needle contacts with brachial plexus nerve fibres eliciting short lasting electric sensations especially when injections are placed too close to Erb's point.

### Arm dystonia

Arm dystonia can be divided into action induced forms and spontaneous forms. Action induced forms occur only during certain activities which can sometimes be highly specific. Spontaneous forms are not associated with specific activities, although they may be increased by unspecific physical activity. Writer's cramp is the most common action induced dystonia (Sheehy et al. 1988). Other highly specific activities can also trigger dystonia, such as playing musical instruments (producing musician's cramps) or performing sports. When they are performed as part of a profession they may be called occupational cramps.

In writer's cramp a wrist flexor and a wrist extensor type can be distinguished. Additionally, elbow and shoulder muscles may be involved. However, abnormal elbow and shoulder postures may be compensatory to change the writing position and to reduce dystonia. Most frequently finger and thumb muscles may be involved. Planning of BT therapy for writer's cramp is based on careful examination of the clinical symptomatology. Electromyography is rarely contributory, since normal writing usually generates wide spread muscle activation which can hardly be distinguished from dystonic muscle activity. Results of BT therapy of writer's cramp are limited, because of narrow therapeutic windows of the potential target muscles (Das et al. 2006). This is a problem especially in the finger extensors. Apart from this, writer's cramp frequently affects a large number of forearm muscles. BT therapy targeting all of these muscles would induce major paretic adverse effects. Additionally, distinction between physiological and dystonic muscle activity and identification of compensatory muscle activity may be difficult. Our experience indicates that even after several modifications of the injection scheme only about half of the patients benefit from BT therapy and continue treatment. Results are better when the finger muscles are not involved, i.e. when the symptomatology is restricted to the wrist or elbow muscles. When finger muscles are involved the outcome is better when individual finger muscles and when finger flexors rather than finger extensors are dystonic. If the symptomatology is restricted to individual finger muscles electromyography possibly with additional electric stimulation may facilitate BT placement. If BT therapy is not successful in treatment of writer's cramp, the patient can shift writing to the contralateral hand. About half of the patients can permanently use the contralateral hand for writing, whereas the other half develops writer's cramp in this hand as well within one or two years. Increased use of keyboards is also one option to circumvent writer's cramp. Re-training exercises may also become a therapeutic option in the future (Zeuner et al. 2002).

Treatment of other action induced arm dystonias, especially when they are occupational, is even more problematic, since the motor performance expected by the patient is usually so high that it cannot be met, either due to dystonic residues or due to therapy induced paresis (Jabusch et al. 2005).

Spontaneous arm dystonia usually occurs as part of a spasticity-dystonia syndrome or as idiopathic dystonia. Typical postures include finger flexion, thumb flexion, wrist flexion, elbow flexion and should adduction or shoulder abduction. In spasticity-dystonia syndrome treatment is focused on pain, prevention of contractures and eased care. Functional improvement may result, but is often restricted by the underlying paresis.

### Leg dystonia

Leg dystonia can occur in idiopathic as well as in symptomatic dystonia, mostly as part of a spasticity-dystonia syndrome due to stroke or cerebral palsy. Action induced forms would be extremely rare. Typical postures include hip adduction, knee flexion, equinovarus posture, i.e. the combination of ankle plantar flexion, foot supination and toe flexion, as well as ankle plantar flexion and toe flexion. Hip adduction is caused by activation of the adductor muscle group (adductor magnus, minimus, longus, brevis and gracilis muscles), knee flexion by activation of the hamstrings (semimembranosus, semitendinosus, biceps femoris muscles) and the equinovarus posture by activation of the tibialis posterior, triceps surae, flexor hallucis and digitorum longus muscles. Ankle plantar flexion is the result of activation of triceps surae, peroneus longus and brevis and flexor digitorum longus muscles, whereas toe flexion is produced by the activation of the flexor digitorum longus and brevis muscles.

Hip adduction, ankle plantar flexion and equinovarus postures respond well to BT therapy. BT doses, however, may be high, especially when bilateral injections are necessary. BT therapy for knee extension bears the risk of knee weakness, especially in patients with additional paresis as in spasticity-dystonia syndrome after stroke. Toe and great toe flexion often requires combined treatment of flexor digitorum brevis and longus muscles.

### Segmental dystonia, generalised dystonia

BT therapy of extended dystonic symptomatologies usually requires selection of those target muscles which play a key role in functional impairment, pain and prevention of complications. Less relevant target muscles may need to be left untreated not to exceed safe total BT doses (Rosales and Dressler 2016). When safety margins are exploited to their full extent, BT therapy can improve even extended symptomatologies substantially. In patients requiring excess BT doses deep brain stimulation may offer a treatment alternative. Combinations of BT therapy and deep brain therapy or intrathecal baclofen are possible.

## Outlook

BT therapy has revolutionised many medical fields. In dystonia, it offers for the first time help to large numbers of patients with focal dystonia. In segmental and generalised dystonia, high BT doses are necessary. The introduction of low antigenicity BT drugs allows reduced inter-injection intervals for an improved dynamic adjustment and increased BT dose for treatment of extended BT symptomatologies. High affinity BT drugs may improve antigenicity even further. They may also reduce the threshold for systemic toxicology so that higher total BT doses may be applied. The biggest challenge for the future, however, is to deliver state of the art BT therapy to all dystonia patients in need regardless whether they live in developed countries or beyond. For this new business models need to be established to allow awareness programs, to provide reimbursement of drug costs and to re-adjust cost-market size calculations to allow prize reductions whilst at the same time providing sufficient funds for market development.
